# Diltiazem Reduces Mortality and Breakdown of ATP in Red Blood Cell Induced by Isoproterenol in a Freely Moving Rat Model *in Vivo* †

**DOI:** 10.3390/metabo4030775

**Published:** 2014-09-11

**Authors:** Pollen K.F. Yeung, Zhaolin Xu, Dena Seeto

**Affiliations:** 1 Pharmacokinetics and Metabolism Laboratory, College of Pharmacy and Department of Medicine, Dalhousie University, Halifax, NS B3H 4R2, Canada; E-Mail: dena.seeto@gmail.com; 2 Department of Pathology, Faculty of Medicine, Queen Elizabeth II Health Sciences Centre and Dalhousie University, 5788 University Ave., Halifax, NS B3H 1V8, Canada; E-Mail: Zhaolin.Xu@cdha.nshealth.ca

**Keywords:** diltiazem, ATP, cardiovascular protection, myocardial infarction, rats, isoproterenol

## Abstract

The benefit of calcium channel blockers for cardiovascular prevention against heart attack and stroke has not been firmly supported. We investigated the possible cardiovascular protective effect of diltiazem (DTZ) against injury induced by isoproterenol using a freely moving rat model *in vivo*. Sprague Dawley rats were injected subcutaneously (sc) with either 5 or 10 mg/kg of DTZ, or saline as control, twice daily for five doses. One hour after the last injection, a single dose of isoproterenol (30 mg/kg) was injected sc to each rat. Blood samples were collected serially for 6 h for measurement of adenine nucleotides (ATP, ADP and AMP) in red blood cell (RBC) by a validated HPLC. The study has shown isoproterenol induced 50% mortality and also increased RBC concentrations of AMP from 0.04 ± 0.02 to 0.29 ± 0.21 mM at the end of the experiment (*p* < 0.05). Treatment with 10 mg/kg of DTZ reduced mortality from 50% to <20% and attenuated the increase of RBC concentrations of AMP from +0.25 ± 0.22 in the control rats to +0.072 ± 0.092 mM (*p* < 0.05). The study concluded that 10 mg/kg of DTZ reduced mortality and breakdown of ATP induced by isoproterenol in rats.

## 1. Introduction

Despite their introduction for clinical use over 30 years ago, calcium channel blockers are still an important class of therapeutic agents widely used for treatment of cardiovascular diseases [1,2,3]. It has been suggested that, in addition to lower blood pressure and heart rate, many calcium channel blockers have inherent anti-oxidant properties which contribute significantly to their cardiovascular protective effect [4,5]. One of the first generation calcium channel blockers is diltiazem (DTZ), which is clinically proven effective for hypertension and angina, and has potential for stroke prevention [6,7]. It is also often used as a probe to study pharmacokinetics and pharmacodynamic interactions with the calcium channel blockers [8,9,10,11]. In addition to lowering blood pressure, DTZ has significant negative chronotropic and inotropic effects, which are important for patients with cardiac arrhythmias and those who need beta-blockers [12]. In addition to the hemodynamic effect, it has been shown that DTZ also suppressed innate immunity and possessed anti-oxidant effect which can protect against tissue injury from reactive oxygen species [13,14].

Previous studies have shown that the cardiovascular effects of DTZ are more sustainable after multiple doses than a single dose [15]. A 5 mg/kg of DTZ given by subcutaneous injection (sc), twice daily for five doses to spontaneously hypertensive rats (SHR) decreased ambulatory blood pressure by as much as 20–30% and heart rate by about 15% [16]. In addition to lowering blood pressure and heart rate, DTZ also inhibits adenosine uptake and metabolism by red blood cell (RBC) which enhances circulatory concentrations of adenosine [17], which may contribute to the anti-ischemia and cardiovascular protective effect [18]. It has been shown that DTZ pretreatment (25 mg/kg/day for 50 days orally) can prevent diastolic heart failure and sudden death induced by isoproterenol in a transgenic mouse model with cardiac hypertrophy [19]. Despite the potential for cardiovascular prevention, there is, as of yet, very little firm evidence to support its effectiveness against myocardial infarction (MI). We have shown previously that acute myocardial injury induced by a single dose of isoproterenol (30 mg/kg) was characterized by an abrupt and profound decrease in systolic and diastolic blood pressures, which rebounded back to pretreatment level shortly (<1 hr) after the injection. The heart rate was increased immediately and stayed elevated throughout the experiment. In addition, isoproterenol also increased RBC concentrations of adenosine 5’-monophosphate (AMP), and those rats did not survive from the acute MI had much higher concentrations of AMP [20]. However the study did not assess possible modulation by pharmacologic agents. The current study is aimed to follow up the previous study and investigate a possible mechanism for the cardiovascular protective effect of DTZ against injury induced by isoproterenol using a freely moving rat model *in vivo* as described previously [20,21].

## 2. Results

Under the described experimental condition, none of the rats died in the Control Group D not receiving isoproterenol (*n* = 11). On the other hand, a single dose of isoproterenol (30 mg/kg) given by sc injection induced 50% mortality in the normal saline treated rats (Control Group C) (*n* = 10) (*p* < 0.05 *vs.* Group D). In the rats treated with 5 or 10 mg/kg of DTZ, twice daily for five doses, by sc injection, the mortality rate was > 60% (four out of six died) and < 20% (one out of six died), respectively. However due to the small sample size in each group, the differences were not statistically significance (*p* > 0.05 *vs.* Control Group C).

**Figure 1:**
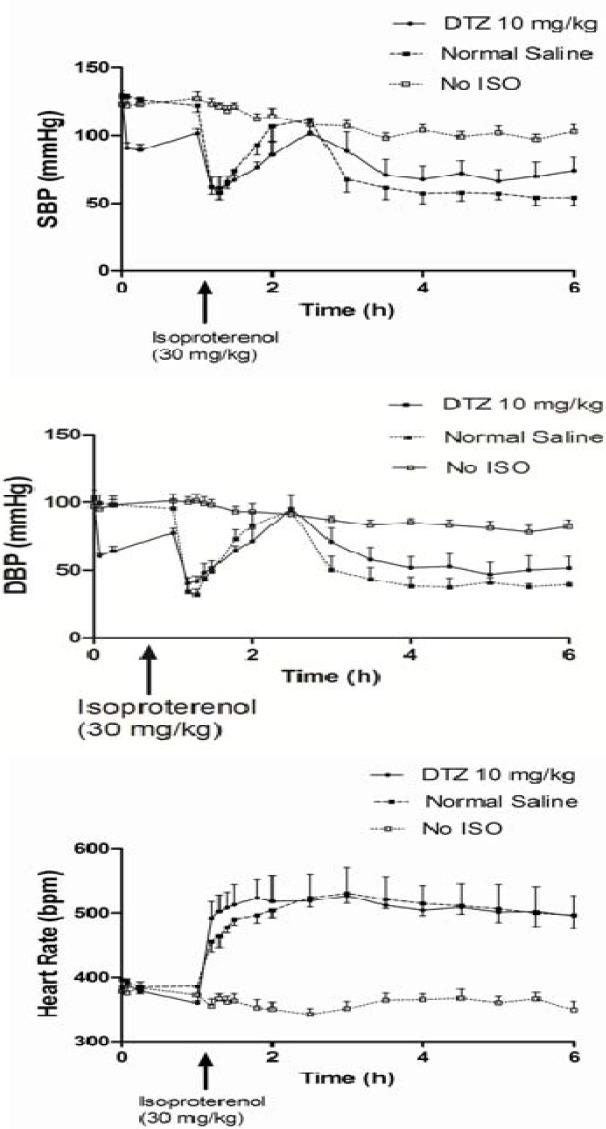
Hemodynamic effect of DTZ in rats treated with isoproterenol (30 mg/kg). Each point represents mean and SEM (*n* = 6 for DTZ 10 mg/kg Group; *n* = 10 for Normal Saline Group; *n* = 11 for No ISO Group). Abbreviations: DBP = diastolic blood pressure; SBP = systolic blood pressure; ISO = isoproterenol; DTZ = diltiazem.

As expected, DTZ lowered blood pressure (both systolic and diastolic) and heart rate immediately following injection (*p* < 0.05 by paired t-test) (Figure 1). The hemodynamic effect reached a maximum in 15 min, and returned to baseline levels before the next injection, as evidenced by the similar hemodynamic parameters between the DTZ treated groups (A and B) before the last injection and those in the control groups (C and D) (Table 1). The blood pressure lowering effect appeared to be greater after the 10 mg/kg dose, but the effect on lowering the heart rate in contrary was greater after the 5 mg/kg injection although only the difference for diastolic blood pressure was significant (*p* < 0.05) between the two doses (Table 1). Following the isoproterenol injection (30 mg/kg), the blood pressure (systolic and diastolic) fell immediately with a corresponding increase in heart rate (Figure 1). There was a rebound of the blood pressure, back to close to pre-treatment levels, within 1–2 h after isoproterenol administration, but the heart rate remained greatly elevated for the remainder of the experiment (Figure 1). As three out of the six rats treated with 5 mg/kg dose of DTZ died within 20 min of isoproterenol administration, to avoid bias, the hemodynamic and biomarker data after isoproterenol in this group were excluded from comparison.

**Table 1:** Cardiovascular effect of DTZ before isoproterenol (Iso) injection in Rats.

Biomarkers/Treatment	A DTZ (5 mg/kg) (*n* = 6)	B DTZ (10 mg/kg) (*n* = 6)	C Control (No DTZ) (*n* = 10)	D Control (*n* = 11) No Iso and DTZ)
SBP (mmHg) before the last dose	130 ± 8^a^	130 ± 8	127 ± 14	123 ± 11
DBP (mmHg) before the last dose	105 ± 12	102 ± 7	104 ± 19	100 ± 15
HR (bpm) before the last dose	371 ± 22	385 ± 32	396 ± 40	378 ± 48
SBP (mmHg) T0.25	98 ± 7*	89 ± 10*	126 ± 14	123 ± 15
DBP (mmHg) T0.25	68 ± 4*^,^**	60 ± 4*	98 ± 21	98 ± 13
HR (bpm) T1	351 ± 32	360 ± 9*	386 ± 33	372 ± 47
AUC of ATP in RBC from T0–T1 (mM*T)	2.18 ± 0.53	1.92 ± 0.16	1.88 ± 0.25	1.64 ± 0.31
AUC of ADP in RBC from T0–T1 (mM*T)	0.33 ± 0.09*	0.32 ± 0.02*	0.46 ± 0.09	0.42 ± 0.15
AUC of AMP in RBC from T0–T1 (mM*T)	0.02 ± 0.01*	0.03 ± 0.01	0.05 ± 0.02	0.05 ± 0.03

^a^Mean ± SD; * *p* < 0.05 *vs*. Group C; ** *p* < 0.05 *vs*. Group B; Abbreviations: DTZ (diltiazem); Iso (isoproterenol); SBP (systolic blood pressure); DBP (diastolic blood pressure); HR (heart rate); RBC (red blood cells); AUC (area under the curve); T0 (Time before the last injection); T0.25 (Time at 15 min after the last injection); T1 (Time at 1 hr after the last injection).

**Figure 2:**
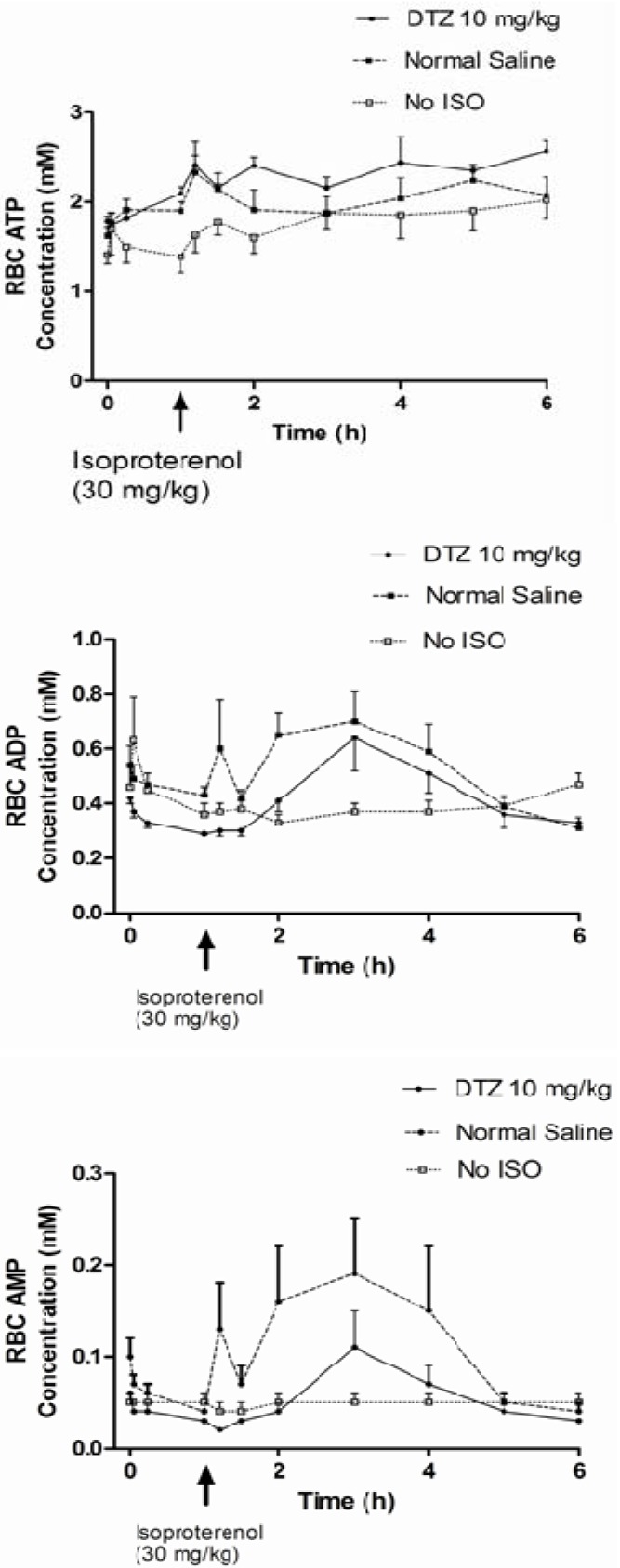
Effect of DTZ on red blood cell concentrations of adenine nucleotides in rats treated with isoproterenol (30 mg/kg). Each point represents mean and SEM (*n* = 6 for DTZ 10 mg/kg Group; *n* = 10 for Normal Saline Group; *n* = 11 for No ISO Group).

Abbreviations: ISO = isoproterenol; RBC = red blood cell; DTZ = diltiazem.

The AUC of ATP concentrations in the RBC from time 0 (T0) to the time before the isoproterenol injection (T1) appeared to be higher while the AUC of ADP and AMP concentrations in the RBC were lower in the rats treated with DTZ, although only the differences in the ADP and AMP concentrations were statistically significant (*p* < 0.05) (Table 1). The concentrations of ADP and AMP increased in the RBC shortly after isoproterenol in both control and DTZ treated rats, and returned to baseline levels towards the end of the experiment (Figure 2). It increased RBC concentrations of AMP from 0.04 ± 0.02 mM before the isoproterenol injection, to 0.29 ± 0.21 mM at the end of the experiment in the control rats (*p* < 0.05), but the increase was not statistically significant in the DTZ treated rats (0.03 ± 0.01 *vs.* 0.10 ± 0.086 mM) (*p* > 0.05). The maximum concentrations of AMP in the RBC after isoproterenol (Cmax) were also significantly higher in the control group C (0.29 ± 0.21 mM) than in the DTZ treated rats (0.10 ± 0.086 mM) and the control group D not receiving DTZ and isoproterenol (0.059 ± 0.030 mM) (*p* < 0.05 Table 2). A similar observation was found when the AUC ratios of AMP to ATP in the RBC were compared (Table 2). There was a tendency of an increase of RBC ATP concentrations towards the end of the experiment, both in the DTZ treated rats (+ 0.43 ± 0.28 mM in Group B) and also in the rats not receiving isoproterenol (+0.63 ± 0.83 mM in Group D) (Figure 2). In comparison, however, there was no increase of the ATP concentrations in the Group C rats, not receiving DTZ (−0.001 ± 0.78 mM) (Figure 2). The difference between the groups, nevertheless, did not reach statistical significance (*p* > 0.05), because of the small sample size and large variation of the data.

**Figure 3:**
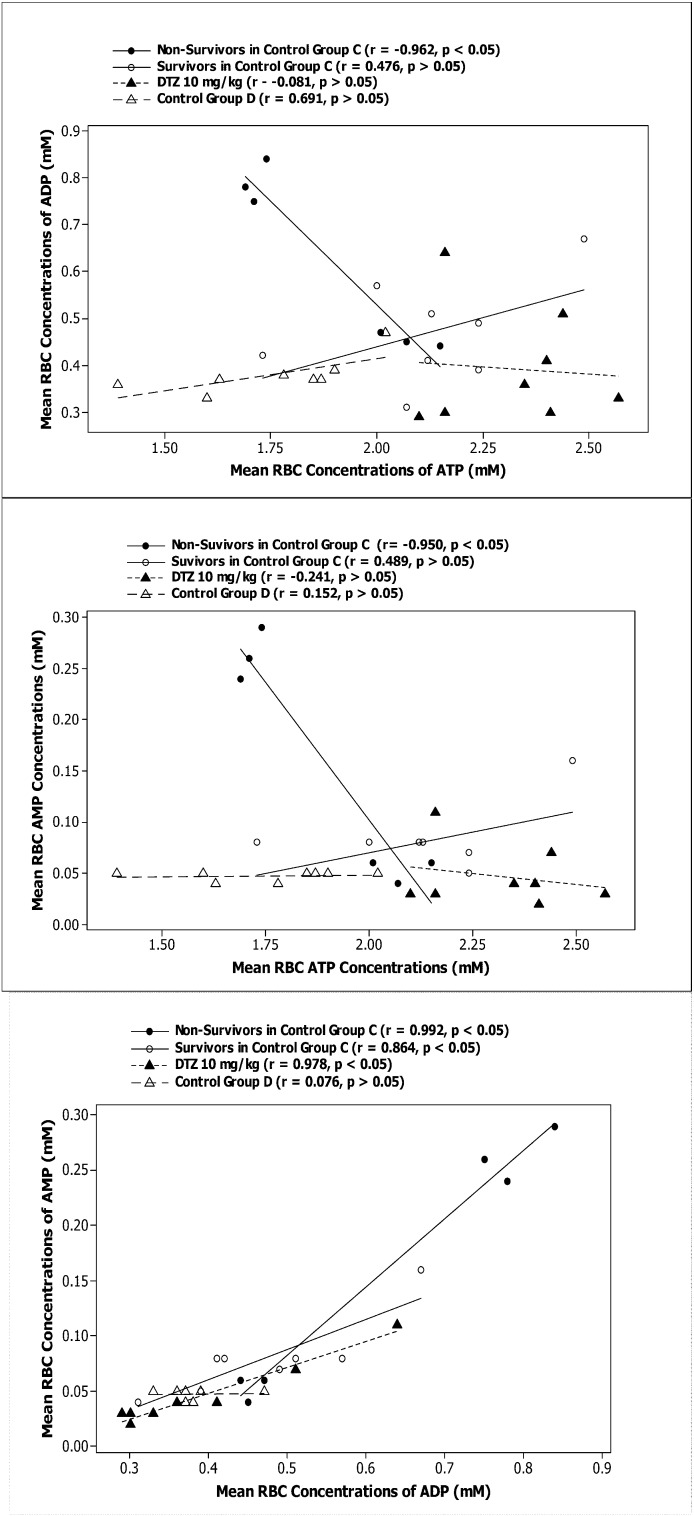
Correlations between mean red blood cell concentrations of adenine nucleotides in rats (*n* = 6 for DTZ 10 mg/kg Group; *n* = 11 for Control Group D; *n* = 5 in the Survivors in Control Group; and *n* = 5 in the Non-Survivors in Control Group). Abbreviations: RBC = red blood cell; DTZ = diltiazem.

Significant correlations (*p* < 0.05) were observed between the mean RBC concentrations of ATP and ADP, and also between ATP and AMP concentrations in the rats which did not survive from the isoproterenol induced injury, but not in the surviving rats, nor in the rats receiving DTZ (10 mg/kg) and those not treated with isoproterenol (Figure 3). Significant correlations were also observed between the mean RBC concentrations of ADP and AMP in the isoproterenol treated rats (survivors and non-survivors) with and without protection from DTZ (10 mg/kg), but not in the rats not receiving isoproterenol (Figure 3). Analysis of individual rat data showed a significant difference (*p* < 0.05) in the correlation coefficient (r) and regression coefficient (β) between RBC concentrations of ATP and AMP comparing the surviving rats and those died (victims) before the end of the experiments (6 hrs after isoproterenol injection). A significant difference was also found for β from regression analysis of ADP and AMP concentrations between the survivors and victims from isoproterenol injection in the rats without protection from DTZ (10 mg/kg) (Table 3). However, there were no significant differences in any of the r or β values between the DTZ treated rats and the control group C (survivors or victims) (Table 3).

**Table 2:** Cardiovascular effect of DTZ after isoproterenol (Iso) injection in Rats.

*Biomarkers/Treatment*	*A* *DTZ (5 mg/kg) (n = 6)*	*B* *DTZ (10 mg/kg) (n = 6)*	*C* *Control (No DTZ) (n = 10)*	*D* *Control (n = 11) No Iso and DTZ)*
SBP (mmHg) immediately before Iso or at 1 hr	110 ± 14^a,^**	102 ± 8*^,^**	126 ± 14	127 ± 15
SBP (mmHg) 10 min after	60 ± 10**	62 ± 17**	62 ± 17**	123 ± 14
Change in SBP (mmHg)	−50 ± 11**	−40 ± 17*^,^**	−64 ± 20**	−3 ± 10
DBP (mmHg) immediately before Iso or at 1 hr	83 ± 8**	77 ± 9*^,^**	95 ± 22	98 ± 13
DBP (mmHg) 10 min after	28 ± 15**	41 ± 10**	34 ± 17**	100 ± 12
Change in DBP (mmHg)	−49 ± 4**	−37 ± 15*^,^**	−61 ± 19**	0 ± 10
HR (bpm) immediately before Iso or at 1 hr	351 ± 32	360 ± 9*^,^**	386 ± 33	383 ± 32
HR (bpm) 10 min after	476 ± 19**	492 ± 62**	456 ± 50**	372 ± 47
Change in HR (bpm)	+120 ± 37*^,^**	+132 ± 66**	+70 ± 53**	−10 ± 29
Change in HR (bpm) at the end of experiment	+149 ± 62**	+175 ± 73**	+157 ± 56**	−14 ± 43
AUC of ATP in RBC from T1 – T last (mM*T)	ND^b^	9.83 ± 4.75	7.74 ± 3.76	9.08 ± 3.03
AUC of ADP in RBC from T1 – T last (mM*T)	ND	2.21 ± 0.45	2.15 ± 0.73	1.85 ± 0.49
AUC of AMP in RBC from T1 – T last (mM*T)	ND	0.29 ± 0.13	0.48 ± 0.37	0.23 ± 0.14
AUC AMP / AUC ATP in RBC from T1 – Tlast	ND	0.02 ± 0.01*	0.09 ± 0.09**	0.02 ± 0.02
Cmax of ATP in RBC (mM) after Iso or 1 hr	2.56 ± 0.53	2.76 ± 0.41	2.42 ± 0.65	2.34 ± 0.70
Cmax of ADP in RBC (mM) after Iso or 1 hr	0.49 ± 0.23*	0.60 ± 0.28	0.88 ± 0.37**	0.50 ± 0.12
Cmax of AMP in RBC (mM) after Iso or 1 hr	0.11 ± 0.13	0.10 ± 0.086*	0.29 ± 0.21**	0.059 ± 0.030

^a^Mean ± SD; ^b^NA = Not applicable; ^c^ND = Not done because of missing data; *p < 0.05 vs Group C; **p < 0.05 *vs* Group D. Abbreviations: DTZ (diltiazem); Iso (isoproterenol); SBP (systolic blood pressure); DBP (diastolic blood pressure); HR (heart rate); RBC (red blood cells); AUC (area under the curve); T1 (Time at 1 hr after the last injection); Tlast (Time when the last sample from the experiment was taken); Cmax (Maximum concentration).

**Table 3:** Correlation between red blood cell concentrations of adenine nucleotides in rats after isoproterenol (Iso).

Biomarkers/Treatment	A DTZ (5 mg/kg) (*n* = 6)	B DTZ (10 mg/kg) (*n* = 6)^c^	C Control No DTZ (*n* = 5) Survivors	C Control No DTZ (*n* = 5) Victims
ATP *vs.* AMP r	ND^b^	−0.308 ± 0.326^a^	0.056 ± 0.409*	−0.721 ± 0.352
ATP *vs.* AMP β	ND	−0.097 ± 0.177	0.023 ± 0.063*	−0.317 ± 0.190
ATP *vs.* ADP r	ND	−0.038 ± 0.571	0.016 ± 0.460	−0.426 ± 0.656
ATP *vs.* ADP β	ND	−0.260 ± 0.534	0.039 ± 0.198	−0.521 ± 0.643
ADP *vs.* AMP r	ND	0.744 ± 0.501	0.708 ± 0.384	0.763 ± 0.290
ADP *vs.* AMP β	ND	−0.014 ± 0.527	0.102 ± 0.104*	0.470 ± 0.282

^a^ Mean ± SD; ^b^ ND = Not done because of missing data; ^c^ Determined from the survivors; * *p* < 0.05 *vs*. Group C Victims; Abbreviations: DTZ (diltiazem); r (Pearson Correlation Coefficient); β (Regression Coefficient).

## 3. Discussion

As shown previously by our laboratory, and others, DTZ has a significant effect lowering blood pressure (systolic and diastolic) and heart rate after both 5 and 10 mg/kg doses although the effects lasted only for several hours [16,22], and had subsided long before the next dose (12 hrs). It is interesting to note that, while the effect of DTZ on lowering diastolic blood pressure was dose dependent, such that the effect was significantly greater following the 10 mg/kg dose (*p* < 0.05) than after the 5 mg/kg dose, there was no significant difference for the effect on systolic blood pressure and heart rate between the two doses (Table 1). The effect of an increase in DTZ dosage from 5 to 10 mg/kg also increased the survival rate from the cardiovascular insult induced by isoproterenol from < 40% to > 80%. However, due to the small number of animal used (*n* = 6 in each group), the difference was not statistically significant (*p* > 0.05). An increase in sample size to 10 animals in the treatment groups could achieve a statistically significant difference. Appreciable cardiovascular protective effect of DTZ has previously been reported against severe diastolic heart failure and cardiac sudden death induced by isoproterenol in a transgenic mouse model after 25 mg/kg/day for 50 days by oral gavage [19]. In addition to the hemodynamic effect, DTZ also decreased concentrations of ADP and AMP significantly in the RBC (*p* < 0.05) and modestly increased ATP concentration (*p* > 0.05) (Table 1) in the rats prior to receiving isoproterenol. We have shown earlier that DTZ also increased RBC concentrations of ATP in a zebrafish model [23] and in an exercise rat model [24], but not in rats kept in a restrainer [25]. The results suggest that the effect of DTZ on ATP metabolism in RBC *in vivo* may be enhanced when energy demand increases in the host species, as in the current study. However, it is not clear whether or not the reduction in mortality in the current experiment was attributed to the decrease in diastolic blood pressure, and/or moderation of ATP metabolism in the RBC or by other yet undetermined factors. A third control group, receiving only DTZ but not isoproterenol, may help to further tease out the cardiovascular protective effect of DTZ

As shown previously, a single dose of 30 mg/kg of isoproterenol given by sc injection in the experimental rat model can induce significant mortality (*ca.* 50%), profoundly altered the cardiovascular hemodynamics, and significantly increased RBC concentrations of AMP. In the rats which could not survive the effects, there was a steeper rebound in systolic and diastolic blood pressures following the immediate fall, and a significant increase in break down of ATP to AMP in the RBC than the surviving rats [20]. It appears that 10 mg/kg of DTZ given twice daily for 5 doses improved survival rate and significantly reduced the AMP concentrations and the AUC ratio of AMP to ATP, which is a measure of catabolism of ATP to AMP in RBC (Table 2). Correlation analyses of the data indicated that the rats, which did not survive from the cardiovascular injury, had significantly more breakdown of ATP in RBC to ADP (*r* = −0.962, *p* < 0.05) and also to AMP (*r* = −0.950, *p* < 0.05). The breakdown of ATP in the RBC was not significant in the DTZ treated group and also in the surviving rats not receiving DTZ (Figure 3 and Table 3). The data also indicated significant positive (+) correlations between RBC concentrations of ADP and AMP after isoproterenol in both control and DTZ treated rats (*r* > 0.85 and *p* < 0.05) (Figure 3). This may suggest that catabolism of ADP to AMP triggered breakdown of ATP to maintain adequate supply of ADP in the RBC in response to cardiovascular injury. This was supported by a significantly greater (+) regression coefficient between ADP and AMP in the rats, which could not survive from the administration (0.470 ± 0.282 *vs*. 0.102 ± 0.104, *p* < 0.05) (Table 3). In these rats, the regression coefficient between ATP and AMP were negative (-) which was significantly different from the survivors (−0.317 ± 0.190 *vs*. 0.023 ± 0.063, *p* < 0.05) (Table 3). The results further support our hypothesis that ATP breakdown to AMP in the RBC *in situ* is a serious cardiovascular event, which may be used as a surrogate for potential mortality.

Consistent with the effect to increase ATP concentrations in RBC during exercise [24], DTZ also appeared to preserve ATP concentrations in RBC after isoproterenol (Figure 2 and Table 2). It is known that, during ischemia (or hypoxia), there is an increase of intracellular calcium ion concentrations and break down of intracellular ATP to release adenosine into systemic circulation. Previous studies from our laboratory and others have shown that calcium channel blockers including DTZ are potent inhibitors of adenosine transport in RBC [26,27]. This could result in an increase in extracellular concentrations of adenosine and preservation of intracellular ATP in the myocardium [28,29,30]. The exact mechanism is unclear although it could be attributed to both blockade of calcium influx from ion channels and release from intracellular calcium storage [14], as well as reuptake and metabolism of adenosine by DTZ and its metabolites, which are known to have similar properties as the parent DTZ [17]. However, the current study could not confirm if RBC concentrations of ATP could be an indicator of the concentrations in the myocardium, and that cardiac tissue damage after isoproterenol was minimized by DTZ, which clearly warrants further investigation.

It has been hypothesized that RBC may serve as an oxygen sensor in the cardiovascular system [31,32]. It was shown that RBC are capable of releasing increased amounts of ATP as oxygen content falls and its hemoglobin becomes desaturated [33]. Thus, when RBC travels through the microcirculation it could release vasodilatory compounds, such as ATP, that enhance blood flow in hypoxic tissues [32]. The released ATP would help to increase blood supply to the tissue and preserve an optimum balance between oxygen supply and demand, thereby modulating the concentrations of tissue ATP within the cardiovascular system. Such a mechanism would eliminate the requirement for a diverse network of sensing sites throughout the vasculature, and should provide a more efficient means of appropriately matching oxygen supply with demand, and allow an immediate switch to alternative energy sources under hypoxia condition [34]. If these hypotheses are proven correct, RBC concentration of ATP may be used as a surrogate marker for cardiovascular protection, and also a pharmacodynamic biomarker for DTZ and other calcium channel blockers, in addition to the hemodynamic effects.

Another important question we could not answer from the study is why the rats that received the 5 mg/kg DTZ dose did not show any improvement in survival compared to the control rats. In fact, three of the four rats died within one hour after isoproterenol in the rats received the 5 mg/kg diltizem dose, as compared to the survival time of three to four hours found in control group C. As the rats died so early in the experiment, we could not collect sufficient data to compare the difference for the hemodynmiac effect and RBC concentrations of adenine nucleotides between the two DTZ doses after isoproterenol. The early mortality could be related to a dose dependent phenomenon, attributing to sympathetic activation and the compensatory mechanism it provokes to maintain cardiovascular homeostasis in response to the injury induced by isoproterenol. It should be noted that the 10 mg/kg DTZ dose administered to the rats is approximately four times the maximum oral dose used clinically for hypertension or angina [35]. Despite the known species difference in pharmacokinetics and pharmacodynamic between human and rats in which first pass metabolism after oral dose is 50 times greater in rats compared to humans [36], we should be cautious of the clinical relevance of the protective effect demonstrated at such a high dose, and that there may be other calcium channel blockers having greater cardiovascular protective effect. Neurohormone and sympathetic activation is an important target for management of cardiovascular diseases [37,38,39]. It has been known for many years that calcium channel blockers differ widely in potency to provoke sympathetic activation [40,41,42]. It is possible that depending on the effect against sympathetic activation, these therapeutic agents may also differ in their protective effects against cardiovascular injury induced by isoproterenol. The implication of the concept of neurohormone (sympathetic) activation in response to isoproterenol and the relationship with the cardiovascular protective effect of DTZ and other cardiovascular agents warrants further investigation.

## 4. Experimental Section

### 4.1. Chemicals

Authentic standards of purine nucleotides including ATP, adenosine-5'-diphosphate (ADP), AMP, and isoproterenol hydrochloride were purchased from Sigma-Aldrich Chem Co. (St. Louis, MO, USA). Solvents were HPLC grade, and all other chemicals were reagent grade (Fisher Scientific, ON, Canada).

### 4.2. Animal Study

The protocol followed the Canadian Council on Animal Care guidelines and was approved by the Dalhousie University Committee on Laboratory Animals (Protocol No 10-003 approved on Mar 1, 2011). Sprague Dawley rats with a carotid artery catheter weighing between 250 to 320 g were used (Charles River Laboratories, Wilmington, MA, USA). They were acclimatized at the Carleton Animal Care Centre with free access to food and water for 48 h before experiment. Each rat was injected subcutaneously with either 5 mg/kg (Group A) or 10 mg/kg of DTZ (Group B), twice daily for five doses (*n* = 6 for each group), which have been shown in previous studies to significantly lower blood pressure and heart rate in a similar rat model [15,16]. A control group (Group C) (*n* = 10) received normal saline (1 mL/kg) in the same regimen. During experiment, each rat was kept in a freely moving caging environment with free access to drinking water as described previously [20]. In the treatment group, one hour after the last injection, isoproterenol hydrochloride (30 mg/kg) freshly prepared in normal saline (30 mg/mL) was administered by subcutaneous (sc) injection in the dorsal area of each rat. A second control group (Group D) (*n* = 11) received normal saline (*i.e.*, no isoproterenol and DTZ) was employed for further comparison. Blood samples (0.3 mL each) were collected from each rat via the indwelling catheter before the last dose (0 hr or T0), and then at 0.05, 0.25, 1, 1.2, 1.5, 2, 3, 4, 5, and 6 hrs after the last dose. The blood samples collected were immediately mixed with a “Stopping Solution” for measurement of adenine nucleotides (ATP, ADP and AMP), which is a mixture of made up of 26 µM EHNA, 100mM dipyridamole, 4 mM EDTA, and a final concentration of 2 μg/mL of indomethacin in heparinized normal saline with pH adjusted to 7.4 to minimize *in vitro* degradation and production of adenosine during sample processing [24]. Hemodynamic recordings including systolic blood pressure, diastolic blood pressure, and heart rate were collected continuously throughout the experiment using a TruWave^®^ disposable pressure transducer (Model PX601, Edwards Lifesciences Canada, Inc., Mississauga, ON, Canada) coupled to a Siemens hemodynamic monitor (Sirecust 400) and chart recorder (Siredoc) (Erlangen, FRG) [43]. The RBC samples collected were processed and lysed immediately using an ice cold 10% trichloroacetic acid. The lysate samples were stored at −80 °C, and concentrations of ATP and other adenine nucleotides in the RBC were determined by a validated HPLC assay [24]. The rats were euthanized at the end of the experiment (6 h after isoproterenol) by cardiac puncture under general anaesthesia with isoflurane.

### 4.3. Data Analysis

Rats which survived longer than six hours from the cardiovascular injury induced by isoproterenol were considered survivors, and those died within six hours were victims. Areas under the curve (AUC) of RBC concentrations of ATP and other adenine nucleotides were calculated using the trapezoidal method (Prism^®^-5, Graphpad Software Inc., La Jolla, CA, USA). Maximum (Cmax) and minimum (Cmin) concentrations of adenine nucleotides and hemodynamic variables were obtained directly from the observed data (Figure 2). Hemodynamic and circulating biomarker variables between the control and isoproterenol treatment groups during the experiment were analyzed by *student’s paired and unpaired t-tests*, and differences considered significant at *p* < 0.05. The Fisher’s exact test was used to assess difference in mortality rate between the treatment groups and considered significance at *p* < 0.05. In addition, possible relationships between biomarkers from the group mean data and individual rat data were assessed using Pearson Correlation and linear regression analyses, and considered significance at *p* < 0.05 (Minitab^®^ Inc., Release 16, State College, PA, USA).

## 5. Conclusions

DTZ (10 mg/kg) significantly reduces hemodynamic changes, RBC concentrations of AMP, and breakdown of ATP in the RBC induced by isoproterenol. There was no protection from 5 mg/kg dose of DTZ.
